# Ethics: use and misuse of assisted reproductive techniques across species

**DOI:** 10.1530/RAF-21-0004

**Published:** 2021-06-18

**Authors:** Madeleine L H Campbell

**Affiliations:** 1 Department of Pathobiology and Population Sciences, The Royal Veterinary College, Hawkshead Lane, South Mymms, UK

**Keywords:** ethics, assisted reproductive techniques, reproduction, veterinary ethics, comparative ethics, reproductive ethics

## Abstract

The boundaries of what we are able to do using ARTs are fast-moving. In both human and veterinary medicine, this presents a fundamental question: ‘Just because we can, should we?’ or, to rephrase the same question: ‘How can we distinguish between what is a use and a misuse of an ART, across species?’ This paper assesses the scientific evidence base for and against the use of ARTs and offers a personal opinion on how we can use such evidence to inform an ethical distinction between justifiable and unjustifiable uses of the techniques. It is argued that the law provides a necessary but insufficient basis for such distinctions. Based on the evidence about harms and benefits, ARTs may be classified into three groups: those which should be rarely used; those for which current evidence supports arguments both for and against their use and those which there is an ethical imperative to use. To which category a particular ART falls into varies depending upon the species to which it is being applied and the reason we are using it. In order to ensure that our ethical oversight keeps up with our technical prowess, the medical and veterinary professions should keep discussing and debating the moral basis of the use of ARTs, not only with each other but also with the lay public.

**Lay summary:**

The use of assisted reproductive techniques (ARTs) has become commonplace in both human and veterinary medicine. Technical limitations are rapidly advancing. This raises a fundamental issue: ‘How can we distinguish between what is a use and a misuse of an ART, across species?’. ‘Misuse’ may be defined both in terms of physical and psychological harms and of moral disquiet about ‘interfering with nature’. This paper assesses the scientific evidence base for and against the use of ARTs and provides a personal opinion on how we can use such evidence to inform an ethical distinction between justifiable and unjustifiable uses of the techniques. We need to consider not only legal but also non-legal ethical justifications for their use. Based on the evidence about harms and benefits, ARTs may be classified into three groups: those which should be rarely used; those for which current evidence supports arguments both for and against their use and those for which there is an ethical imperative to use. To which category a particular ART falls into varies depending upon the species to which it is being applied and the reason we are using it. Open discussion between the medical and veterinary professions and the public is necessary to ensure that ethical oversight of the use of ARTs across species keeps up with technical developments.

## Introduction

We live, as the programme for the recent Fertility 2021 conference evidenced, in an extraordinary age where assisted reproductive techniques (ARTs) enable us not only to freeze gametes for future use, or to perform fertilisation *in vitro* using a single injected sperm (O’Neill *et al.* 2018) but to fashion embryos from component gamete parts of three individuals (Craven *et al.* 2020) and even edit the genome of embryos in ways which will permanently alter hereditary genetic material (West & Gill 2016, Government of Argentina 2018, Campbell & McNamee 2020). The boundaries of what we are technically able to do are fast-moving, as Prof Clark’s extraordinary presentation on *in vitro* gametogenesis at Fertility 2021 demonstrated.

In both human and veterinary medicine, this presents us with a very fundamental question: ‘Just because we can, should we?’ or, to rephrase the same question: ‘How can we distinguish between what is a use and a misuse of an ART, across species?’ ‘Misuse’ may be defined both in terms of physical and psychological harms and of moral disquiet about ‘interfering with nature’.

This paper assesses the scientific evidence base pertaining to the use of ARTs and provides a personal opinion on how we can use such evidence to inform an ethical distinction between justifiable and unjustifiable uses of the techniques. I shall argue that the law is a necessary but insufficient basis for such distinctions. We need to look beyond what the law permits and to interrogate the fundamental ethical basis on which ART ‘treatment’ is accepted by society, and then to consider which factors are important when distinguishing between use and misuse in that context. I shall argue the technical nature of the ART; the purpose for which the ART is being used; an imperative to refine ARTs to minimise harms and interspecies differences are all relevant to such distinction-drawing. In making these arguments, I shall suggest that ARTs may be classified into three groups: those which should be rarely used; those for which current evidence supports arguments both for and against their use and those for which there is an ethical imperative to use.

## The law

The law provides one way of distinguishing between the use and misuse of ARTs. Regulation of new techniques was a repeated topic during Question and Answer sessions at Fertility 2021. In human medicine in the UK, the Human Fertilisation and Embryology Act (2008, as amended) (HFE Act) determine what are legitimate, socially acceptable uses of ARTs for both clinical and research purposes. This legislation constantly evolves as techniques develop and in consultation with public opinion. In veterinary medicine, no such legal definition exists. The use of ARTs is regulated through the Veterinary Surgeons Act (1966) (VSA) and/or the Animal Welfare Act (2006) (AWA) if they are being performed for clinical reasons, and through the Animals (Scientific Procedures) Act (1986) (A(SP)A), if they are being performed for research purposes. Unlike the A(SP)A neither the VSA nor the AWA makes any mention of pre-natal animal forms, meaning that they are offered no protection in relation to clinical procedures. Further, the manipulation of an animal embryo outside of an animal body is apparently unregulated by any of the three Acts, whether it be performed by Vets or non-Vets. This is in striking contrast to human medicine, where the HFE Act specifically protects human embryos outside of the body.

Legislation thus fails through omission to fully determine what is a legitimate use and what a misuse of an ART in non-human animals. Even in the world of human medicine though the law provides one, necessary basis for decision-making about ARTs that is insufficient: the fact that an ART is prohibited in law renders it unethical, but just because something is legal does not necessarily make it ethically acceptable. In order to comprehensively distinguish between the uses and misuses of ARTs, we need to interrogate not only legal but also non-legal ethical justifications for using such techniques.

## Basic ethical justifications for using ARTs

To distinguish between the uses and misuses of ARTs, we need to first understand the fundamental ethical basis on which ART ‘treatment’ is accepted by society at all. Why are we undertaking ARTs, and what are our ethico-social justifications for doing so?

Across species, the fundamental justification for allowing the use of ARTs is a utilitarian one. In human medicine, those requesting the ART procedure anticipate benefiting from it, despite the knowledge of associated harms such as invasive and painful procedures and risks associated with being pregnant and giving birth. The perceived, anticipated psychological and social benefits of being able to have children – in ethical terms to ‘express reproductive autonomy’ – are believed by patients, medics, and society to outweigh (potential) risks.

In veterinary medicine, though the fundamental ethical basis for allowing the use of ARTs is also a utilitarian one, there is a key difference. For non-human animals as for humans, ART procedures and pregnancy carry associated harms. However, in contrast to humans, subfertility apparently has no adverse psychological or societal impact upon an animal. Whilst animals which nuture their young may experience oxytocin-mediated positive emotions as a result of doing so, the absence of that opportunity is probably not something of which the animal is aware, nor which has a negative impact on its quality of life. Thus the benefit of an ART is invariably not to the animal being ‘treated’. It is often instead a predominantly economic (sometimes emotional) benefit enjoyed by the person controlling the animal. It is sometimes a (perceived) benefit to an animal group and even an ecosystem (e.g., where ARTs are being used in an attempt to mitigate against the decline of a species). Thus whilst the fundamental ethical basis on which the use of ARTs in both human and veterinary medicine is permitted by society is a utilitarian one, in veterinary medicine, that justification is predicated on the assumption that we are prepared either to allow human benefits to trump animal harms (Singer 1989, 2009) or to allow benefits at an animal group/species level to trump harms to individual animals. I argue below that the latter justification for the use of ARTs is not defensible where alternative methods which do not inflict harm upon individuals (such as habitat conservation) are available.

In both human and veterinary medicine, utilitarian justifications for the use of ARTs are qualified by ‘respect for nature’ elements. For example, one investigation (Gjerris *et al.* 2006, p. 8) into public attitudes to animal cloning found that people were concerned about ‘violation of the integrity of animals that cloning might constitute’, and that ‘cloning, seems to cross an invisible border between the natural and the unnatural’. In human medicine, ‘respect for nature’ aspects also incorporate sentiments about the integrity and moral status of a human embryo, which date from the time of the Warnock report (Warnock 1984).

## Distinguishing between the use and misuse of ARTs

Having established that societal acceptance of ARTs at all is based predominantly on utilitarian ethics qualified by ‘respect for nature’ elements, I am now going to consider which factors are important when distinguishing between use and misuse on such a basis, across species. I shall argue that the distinction between use and misuse depends upon: (1) the technical nature of the ART, (2) the purpose for which the ART is being used, (3) an imperative to refine ARTs to minimise harms, and (4) interspecies differences. Where the distinction between use and misuse is made with respect to these criteria, ARTs may be classified into those which should be rarely if ever used; those for which current evidence supports convincing arguments both for and against their use; and those for which there is an ethical imperative to employ.

### The technical nature and purpose of the ART

Distinction between use and misuse is easier to make when the ART is either extreme or simple in terms of its technicality and purpose. Whole animal somatic cell nuclear transfer (‘reproductive cloning’) provides an example of an ART which is extreme in both regards. Despite some dissenting opinions (Petersen 2002, Savulescu 2005), the overwhelming worldwide consensus remains that the use of reproductive cloning in humans would be a misuse of ARTs. This moral consensus is based to a large extent in a respect to nature ethics sentiment that cloning is just a step too far (Shapiro 1996, Petersen 2002). More particularly, opposition to human cloning lies in concerns about individual and genetic identity, societal effects, instrumentalisation, eugenic selection (Hope *et al.* 2008, p. 131) and, above all, safety (BMA 1999, Jackson 2010, p. 819). Many of these concerns do not apply to non-human animals. However, the safety argument does. Despite improvements in technology and despite variations across species, it remains the case that cloning of non-human animals is associated with higher rates of placental abnormalities, foetal abnormalities, dystocia, perinatal mortality and need for intensive care, and systemic abnormalities than any other ART (Shapiro 1996, Kuhholzer-Cabot & Brem 2002, Chavatte-Palmer *et al.* 2004, Hinrichs 2005, Constant *et al.* 2006, Loi *et al.* 2006, EFSA 2010, Johnson *et al.* 2010). When one balances these harms to current (including recipient) animals and future generations of animals against the anticipated benefits of many reasons for cloning – for example, the recreation of a favourite pet, or of a successful castrated competition horse in an entire form so that it can be used for breeding – the harms to animals outweigh the relatively trivial benefits to humans. Even when the benefits of cloning are purported to animals – for example, using cloning to conserve endangered species (Williams *et al.* 2006, Fatira *et al.* 2019) – cloning may actually be a misuse of an ART. Well-meaning as such conservation efforts may be, harms to the animals involved are currently unavoidable. Combined with the expense of cloning, this in my view indicates that rather than resorting to harm-causing technologies, humankind should focus finance, research, and policy on addressing underlying issues such as loss of habitat.

However, if one allows the principle that significant human benefits may be allowed to trump animal harms, cloning animals to promote human health may be justifiable. Cloning pigs in which genes had previously been knocked out to reduce the possibility of organ rejection in order to provide a source of heart valves for human transplantation, for example, in this type of analysis provides a sufficiently significant benefit to outweigh the unavoidable harms. Thus the purpose for which the ART is being used as well as the technical nature of the ART itself is important in distinguishing between the uses and misuses of ARTs. Cloning, with its technical challenges and adverse welfare effects, provides an example of an ART which should be rarely if ever used but which, depending on the purpose, might sometimes be ethically permissible.

There are many ARTs, however, for which the distinction between use and misuse is harder to make because current scientific evidence supports convincing utilitarian arguments both for and against their use. One such example is oocyte retrieval and IVF, across species, in relation to the age of the oocyte donor. In human medicine, the use of IVF in older women is controversial. This is partly due to the acknowledged higher rates of harm associated with pregnancy in older women such as gestational diabetes, hypertension, antepartum haemorrhage, pre-term birth, and low birth weight. Some concern also relates to possible negative psychological impacts upon parents and the children. A ‘respect for nature’ sentiment additionally suggests that there is a physiological age beyond which childbearing is not ‘meant to happen’. Many would argue that these harm-based concerns combine to make the use of oocyte retrieval followed by IVF from older women a misuse of such technologies, even when it is requested by the woman.

In horses, to take a veterinary example, a similar situation exists. There is a strong economic imperative to achieve pregnancies from older broodmares with successful progeny. However, older broodmares are more prone to catastrophic disasters during pregnancy, such as prepubic tendon rupture. Placental function is likely to be reduced compared to younger mares (Wilsher & Allen 2003) with adverse consequences upon foetal growth and potentially upon longer-term post-natal health and welfare (Peugnet *et al.* 2014). Anecdotal evidence suggests that oocyte retrieval is itself a harmful, invasive, uncomfortable process for a mare, and the oocytes of older mares are in any case of reduced fertility (Carnevale *et al.* 2005, Rizzo *et al.* 2019). The harm associated with a procedure relates not only to the level of harm but also to the frequency with which that harm is applied. Oocyte retrieval as a precursor to IVF or ICSI often requires repeated cycles of hormonal manipulation and repeated oocyte recovery attempts. These factors support a utilitarian argument against the use of oocyte retrieval and IVF or ICSI in older non-human animals as in humans since it is harmful and has a low success rate.

However, an alternative point of view, also predicated upon a utilitarian analysis, is that the same ART (oocyte retrieval and IVF or ICSI) could be used in conjunction with surrogacy to significantly reduce potential harms whilst facilitating the psychological/social desire of older women to have a family and of mare owners to capitalise on their investments. In women, this could be achieved through oocyte donation from a young donor, IVF using the older woman’s partner’s sperm, and transfer of the resultant embryo to a young surrogate mother. In mares, oocyte retrieval, ICSI, and transfer of the embryo to a young mare’s uterus could enable us to both remove the risk to older mares of carrying a pregnancy and to safeguard the welfare of their offspring. It is thus debatable whether the use of oocyte retrieval and IVF constitutes a misuse or an ethically permissible use of ARTs. Part of our inability to resolve that debate in veterinary species at least is due to sparse evidence about short- and long-term harms associated with ARTs.

Fortunately, distinguishing between use and misuse is not always so problematic. Semen freezing and storage provide an example. In humans, there are clear benefits of being able to freeze semen – for example, facilitating reproduction after cancer treatment, or social reproductive autonomy. In non-human animals, the ability to freeze and transport semen is not only economically advantageous to humans but also means that animals need not themselves undergo the stress and disease risks of transportation. Across species, it appears that the benefits to the wellbeing and welfare of freezing semen outweigh the harms, and it is thus relatively straightforward to define that ART as being ethically permissible rather than misuse.

## The imperative to refine ARTs to minimise harms

Such distinctions, however, are predicated on an ethical imperative to refine ARTs to minimise harm. In the example of semen freezing above, harms are minimal ifnon-invasive methods of semen collection are used. This is commonly the case for men and for species such as horses, dogs, cattle, and sheep. However, invasive methods of semen collection such as *per rectum* electro-ejaculation of rams and bulls do exist. Should these be used in physiologically normal animals in preference to more time-consuming methods of training the animal for non-invasive semen collection that would constitute an abuse of an ART because unnecessary harms are being inflicted.

## Interspecies differences

Sometimes, the same ART may be considered a misuse in one species yet ethically permissible or even required in another. Sex selection provides an example. Currently, two established methods offer the opportunity for sex selection across species: (1) sex sorting of sperm by flow cytometry, and (2) pre-implantation genetic diagnosis of the sex of an embryo (PIGD) (REF).

In the UK, sex selection for non-medical, ‘social reasons’ − for example, simply because the parent(s) prefer to have a child of one sex − is specifically prohibited by the HFE Act. When the HFEA consulted on this issue in 2003, about 80% of respondents felt that social sex selection would be a misuse of ARTs. The public outcry surrounding an investigation into whether this practice was, in fact, being facilitated by British doctors as recently as 2018 suggests that this is still the majority public opinion (Anon 2018).

ln other species, in contrast, there are strong ethical reasons torequirethe use of sex selection for non-medical purposes. Each year, just under 100,000 male dairy calves are killed at a few days old on farms in the UK because it is uneconomic to raise them. Their lack of value may expose them to poor welfare. Recently, there have been admirable attempts to ensure that male dairy calves have good, if short, lives by developing a market for ‘rose veal’. But demand for rose veal is unlikely to equate to the numbers of male dairy calves born each year. Even accepting that sperm sorting is well below 100% accurate, this ethical and welfare issue can and should be significantly reduced by using sex sorting of bovine sperm to select against male calves.

## Conclusion

This paper gives a personal view on how the current scientific evidence base and harm: benefit analysis qualified by ‘respect for nature’ elements can be used to direct ethical decision making about what is an acceptable use and what a misuse of an ART, across species. Such ethical analysis highlights interspecies differences – for example, reproductive autonomy is an important concept in human medicine, whilst it is unlikely to affect the wellbeing of animals. In veterinary medicine, conflicts may exist between the interests of an individual animal (who could be harmed by the use of an ART) and of a species (whose preservation might depend upon such use). The relative weighting which society gives to individuals, to groups, to humans, and to animals is an important aspect of ethical decision-making around ARTs, as are views about what is a morally acceptable level of ‘interference with nature’. Technical capability to manipulate reproduction at a gamete and genetic level is very likely to develop apace over the next 5 years. In order to ensure that our ethical oversight keeps up with our technical prowess, an on-going, open debate about the moral basis of the use of ARTs is needed. Such debate should take place not only within academia and between the medical and veterinary professions and their regulators but also through discussion with the lay public via stakeholder events and consultations.

## Declaration of interest

The author declares that there is no conflict of interest that could be perceived as prejudicing the impartiality of this commentary.

## Funding

This research did not receive any specific grant from any funding agency in the public, commercial or not-for-profit sector.

## Author contribution statement

M C conceived and wrote the paper.
